# Puerperal Diabetes

**Published:** 1883-08

**Authors:** 


### Puerperal Diabetes.

Dr. J. Matthews Duncan contributes a most valuable paper* on this very rare disease. In it he gives histories comprising twenty-two pregnancies in fifteen women, varying in age from twenty-one to thirty-eight years of age. So far as is known all, with one exception, were multiparæ. Of the twenty-two pregnancies in fifteen mothers four ended fatally after delivery. Hydramnios was frequent, and the liquor amnii in one case contained 0.7 per cent. of sugar, and in a second case there was also unquestionably sugar in the liquor amnii, although no

* Transactions of the Obstetrical Society of London, vol. xxiv., p. 256.

quantitative examination was made. In seven of nineteen pregnancies, in fourteen mothers, the child died during the pregnancy, having in all of these reached a viable age. In two more the child was feeble, and died a few hours after birth. In one other case the child had diabetes. The dead fœtus was very large in many of the cases.

Dr. Duncan admits that the number of cases which he has been able to find is too small to possess any very great statistical value, but the histories seem to show—

(1.) Diabetes may come on during pregnancy.

(2.) Diabetes may occur only during pregnancy, being absent at other times.

(3.) Diabetes may cease with the termination of pregnancy, recurring some time afterwards.

(4.) Diabetes may come on soon after parturition.

(5.) Diabetes may not return in a pregnancy occurring after its cure.

(6.) Pregnancy may occur during diabetes.

(7.) Pregnancy and parturition may be apparently unaffected in its healthy progress by diabetes.

(8.) Pregnancy is very liable to be interrupted in its course, and probably always by the death of the fœtus.—*The Boston Medical and Surgical Journal.*

